# Velocity Time Integral (VTI) Measurement of the Coronary Sinus Using Transesophageal Echocardiography During On-Pump Coronary Artery Bypass Graft (CABG) Is Useful for Assessing Treatment Effectiveness: A Case Report

**DOI:** 10.7759/cureus.108610

**Published:** 2026-05-10

**Authors:** Keisuke Sumii

**Affiliations:** 1 Anesthesiology, Saitama Sekishinkai Hospital, Kawagoe, JPN

**Keywords:** cabg, cs, pi, pulse doppler, tee, vti

## Abstract

In on-pump coronary artery bypass grafting (CABG) and left ventricular reconstruction, velocity time integral (VTI) measurement of the coronary sinus (CS) using transesophageal echocardiography (TEE) was useful for evaluating the therapeutic effect of coronary artery reconstruction and recovery of cardiac function. In a 56-year-old male with severe ischemic cardiomyopathy, CS VTI increased significantly from 7.06 cm at induction of anesthesia to 12.48 cm before chest closure, a favorable graft pulsatility index (<3) and improved myocardial perfusion were observed alongside. In this case, we report that the use of VTI measurement of the coronary sinus during on-pump CABG as an intraoperative assessment tool can aid in evaluating the therapeutic efficacy of coronary perfusion improvement.

## Introduction

Patients with severe coronary artery disease often exhibit significant calcification at the coronary artery ostia, making blood flow measurement via transesophageal echocardiography (TEE) difficult. Conversely, the coronary sinus (CS) is relatively easy to identify, and its flow velocity can be measured readily regardless of calcification [1]. The coronary veins run along the groove between the left atrium and the left ventricle on the posterior side of the heart and drain into the CS, located on the posteroinferior aspect of the right atrium [2]. The opening of the CS is approximately 1 cm in diameter and is situated on the inside of the inferior vena cava opening [2]. In addition to the pulsatility index (PI), commonly used for flow assessment during coronary artery bypass grafting (CABG), TEE-based velocity time integral (VTI) measurement of the CS is a simple and semi-invasive test. PI is a key indicator used to assess the function of a newly created graft and the quality of the anastomosis in real time during CABG [3]. Generally, a lower PI indicates better graft patency, and a PI < 3.0 indicates an excellent condition. Conversely, a PI > 5.0 strongly suggests graft complications such as anastomotic kinking, stenosis, or thrombosis [4,5]. However, while the negative predictive value of PI is high, its positive predictive value is not, so it is insufficient on its own as a basis for determining the therapeutic efficacy of surgery [6,7]. VTI is calculated by tracing the waveform of blood flow velocity using the Doppler method and determining its area. It represents the distance traveled by blood through a specific region during a single systole. By multiplying this value by the cross-sectional area of the vessel, it is used to calculate stroke volume (SV) and cardiac output (CO). In coronary angiography (CAG), blood flow measurement is used to assess coronary reserve and evaluate left ventricular perfusion [8]. Studies have reported that measuring VTI in the CS using TEE during on-pump CABG results in increased coronary perfusion in patients with normal left ventricular wall motion. However, the utility of this approach in patients with abnormal left ventricular wall motion has not yet been reported. In this report, we present a case in which we evaluated increased coronary perfusion based on VTI measurements of the CS in a patient with abnormal left ventricular wall motion, and this assessment aided in determining the therapeutic efficacy of CABG [1,9].

## Case presentation

This case involved a 56-year-old male, 171 cm, 52 kg. While undergoing diabetes management at our hospital, he developed exertional dyspnea. On arrival, his vital signs were recorded: he was conscious, with a heart rate (HR) of 104 beats per minute (sinus rhythm), a blood pressure (BP) of 106/65 mmHg, an SpO₂ of 99% on room air, and a respiratory rate of 16 breaths per minute. CAG revealed stenosis at #1 100%, #3 90%, #7 90%, #9 90%, and #13 100%. SYNTAX score was 30.5. Preoperative trans-thoracic echocardiography (TTE) revealed a left ventricular ejection fraction (LVEF) of 20 %, and the impairment of inferior and septal left ventricular wall motion. Left ventricular enlargement was also noted, with a diastolic left ventricular diameter of 55 mm and a systolic left ventricular diameter of 48 mm. Moderate functional mitral regurgitation with a 5 mm eccentric jet directed toward the posterior left atrial wall was present. CABG, mitral valve plasty (MVP) and left ventricular linear closure were performed.

**Figure 1:**
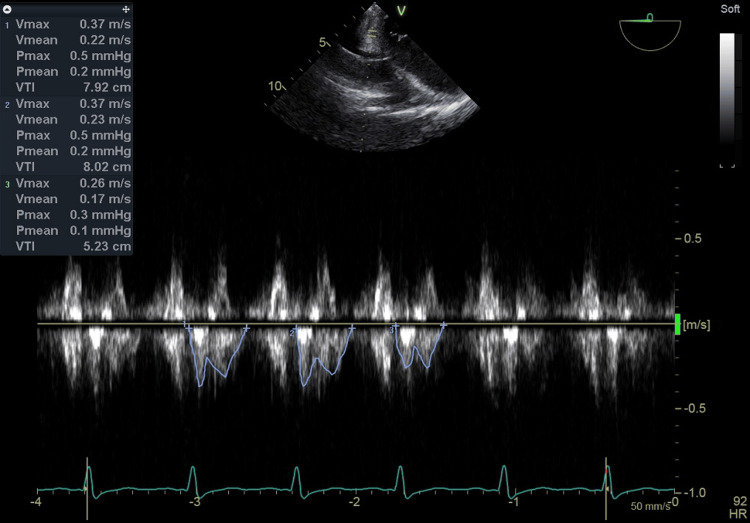
The VTI of CS blood flow measured by the TEE pulse Doppler after induction of anesthesia The VTI value after induction of anesthesia was 7.06 cm (HR 92 beats/min) calculated as the average of three heartbeats excluding premature contractions. In addition, the TEE setting was set to Frames per second 9, Frequency 3.1 MHz, Power 0 dB, Gain -2 dB, Velocity scale 2.0 m/s, Sample volume 4.0 mm, Sample volume depth 2.5 cm and Sweep speed 50 mm/s. VTI: velocity time integral, CS: coronary sinus, TEE: transesophageal echocardiography

**Figure 2:**
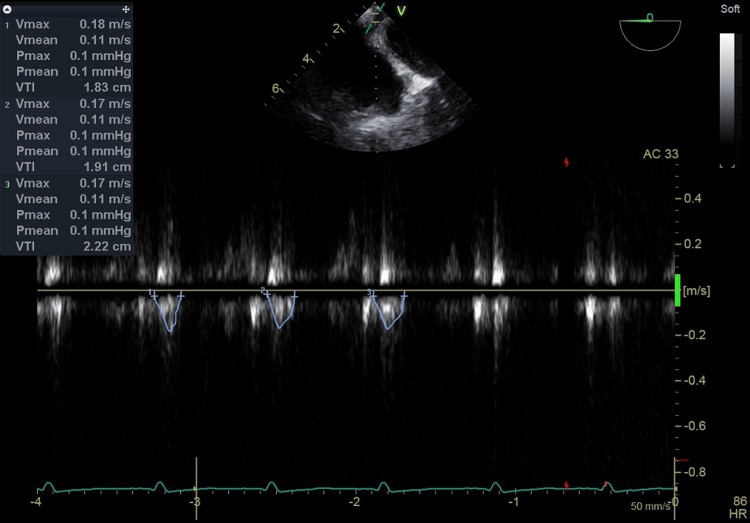
The VTI of CS blood flow measured by the TEE pulse Doppler during heart displacement The VTI value during heart displacement was 4.80 cm (HR 86 beats/min) calculated as the average of three heartbeats excluding premature contractions. In addition, the TEE setting was set to Frames per second 9, Frequency 3.1 MHz, Power 0 dB, Gain -2 dB, Velocity scale 1.5 m/s, Sample volume 4.0 mm, Sample volume depth 0.7 cm and Sweep speed 50 mm/s. VTI: velocity time integral, CS: coronary sinus, TEE: transesophageal echocardiography

**Figure 3:**
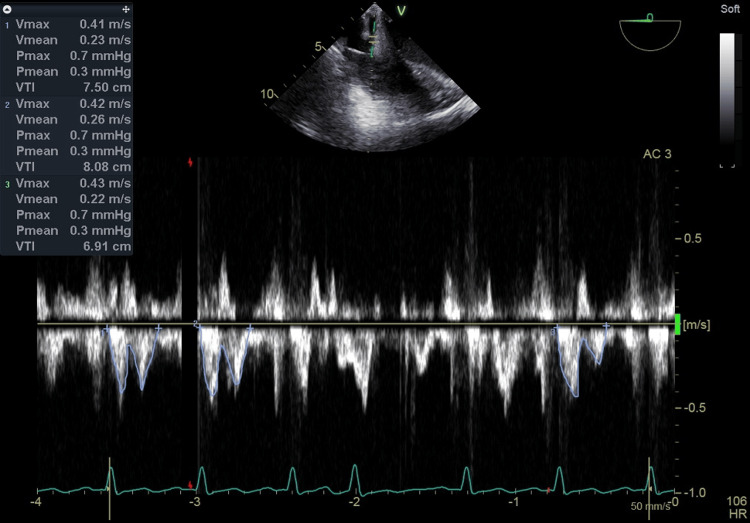
The VTI of CS blood flow measured by the TEE pulse Doppler immediately after weaning from CPB The VTI value after weaning from CPB was 7.50 cm (HR 106 beats/min) calculated as the average of three heartbeats excluding premature contractions. In addition, the TEE setting was set to Frames per second 9, Frequency 3.1 MHz, Power 0 dB, Gain -2 dB, Velocity scale 2.0 m/s, Sample volume 4.0 mm, Sample volume depth 2.7 cm and Sweep speed 50 mm/s. VTI: velocity time integral, CS: coronary sinus, TEE: transesophageal echocardiography, CPB: cardiopulmonary bypass

**Figure 4:**
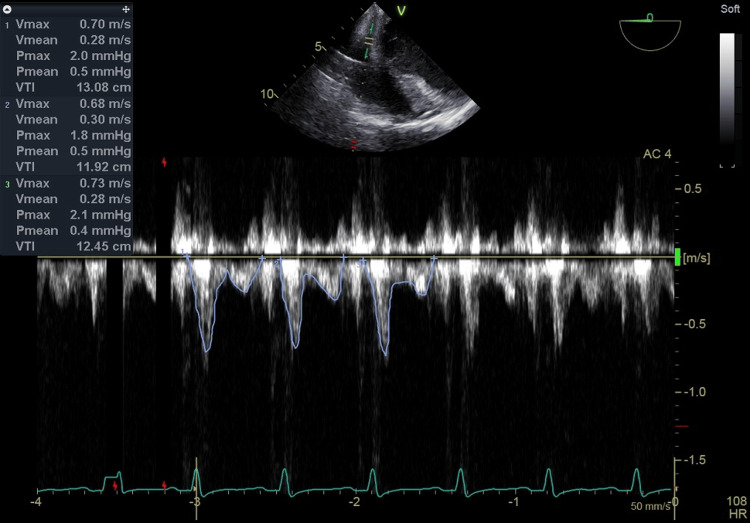
The VTI of CS blood flow measured by the TEE pulse Doppler before chest closure The VTI value before chest closure was 12.48 cm (HR 108 beats/min) calculated as the average of three heartbeats excluding premature contractions. In addition, the TEE setting was set to Frames per second 9, Frequency 3.1 MHz, Power 0 dB, Gain -2 dB, Velocity scale 2.5 m/s, Sample volume 4.0 mm, Sample volume depth 2.8 cm and Sweep speed 50 mm/s. VTI: velocity time integral, CS: coronary sinus, TEE: transesophageal echocardiography

General anesthesia was induced with 50 mg of rocuronium, followed by 8 mg of midazolam and 0.1 mg of fentanyl. Anesthesia was maintained with 1-1.5% sevoflurane and continuous infusion of remifentanil at 0.13-0.26 mcg/kg/min. CABG was performed as follows: left internal mammary artery to left anterior descending branch; aortic root to diagonal branch via great saphenous vein; aortic root to posterior lateral branch via dissected right internal mammary artery; and gastric artery to posterior descending branch. The PIs of the left internal thoracic artery, right internal thoracic artery, great saphenous vein, and gastroepiploic artery after CABG were 1.85, 1.43, 2.29, and 1.89, respectively. After induction of anesthesia, during heart displacement, immediately after weaning from cardiopulmonary bypass (CPB), and before chest closure, the VTI of CS blood flow was measured using the TEE (GE HealthCare Japan, Vivid 95) pulse Doppler. The sample volume was placed approximately 10 mm from the right atrial opening toward the CS in the inferior esophageal CS view. The VTI measurement of CS blood flow was calculated as the average of three heartbeats excluding premature contractions. The mean VTI values for each time point were 7.06 cm for after induction of anesthesia, 4.80 cm for during heart displacement, 7.50 cm for immediately after weaning from CPB, and 12.48 cm for before chest closure (Figures 1-4). The surgery lasted six hours and 58 minutes, anesthesia lasted eight hours and one minute, CPB lasted one hour and 38 minutes, and aortic cross-clamping lasted 36 minutes.

The table shows the LVEF, CO, cardiac index (CI), VTI of CS, BP, HR, VTI×HR/CO, central venous pressure (CVP) and catecholamine doses at each time point (Table 1). MVP and left ventricular reconstruction were performed under CPB. For the mitral valve, ligate the edge of A2 to P2, and approximately 5 cm of the non-contractile area of the left ventricle around the insertion of the posterior papillary muscle was resected to perform left ventricular reconstruction. The left ventricular wall motion of the inferior and septum improved, and resolution of mitral regurgitation was confirmed.

**Table 1:** The table shows the LVEF, CO, CI, VTI, SBP/DBP, MAP HR, VTI×HR/CO, CVP, and catecholamine doses at each time point The LVEF was assessed by eye ball. The VTI of CS blood flow was measured using the TEE pulse Doppler. The VTI measurement of CS blood flow was calculated as the average of three heartbeats excluding premature contractions. Following CABG, the LVEF increased compared to the period after induction of anesthesia. Regardless of the increase in catecholamine dosage or the effects of CO, coronary perfusion before chest closure increased prior to the time of after induction of anesthesia. LVEF: left ventricular ejection fraction, CO: cardiac output, CI: cardiac index, VTI: velocity time integral, SBP/DBP: systolic/diastolic blood pressure, MAP: mean arterial pressure, HR: heart rate, CVP: central venous pressure, CS: coronary sinus, TEE: transesophageal echocardiography, CPB: cardiopulmonary bypass, CABG: coronary artery bypass grafting

	LVEF (%)	CO (L/min)	CI (L/min/m2)	VTI (cm)	SBP/DBP (mmHg)	MAP (mmHg)	HR (beats/min)	VTI×H /CO (cm/L)	CVP (mmHg)	Noradrenaline (mcg/kg/min)	Dobutamine (mcg/kg/min)	Milrinone (mcg/kg/min)
After induction of anesthesia	20	5.4	3.38	7.06	129/65	86	92	120.28	8	0.07	1	-
During heart displacement	-	3	1.88	1.99	104/52	69	86	57.05	5	-	-	-
Immediately after weaning from CPB	-	3.8	2.38	7.5	76/48	57	106	209.21	22	-	-	-
Before chest closure	30	3.6	2.25	12.48	110/58	75	108	374.4	3	0.1	3	0.27

Postoperatively, the patient recovered smoothly without ECG changes or heart failure onset, achieving activities of daily living (ADLs) allowing generally unproblematic daily living. On postoperative day 5, TTE revealed an LVEF of 27%, circumferential left ventricular wall motion impairment, and trivial mitral regurgitation. On postoperative day 8, a coronary CT angiography confirmed patency in all grafts and revealed no abnormalities. On postoperative day 16, the patient was discharged walking unaided without sequelae.

## Discussion

The CS is located near the inferior vena cava in the right atrium and represents the opening where the coronary veins, which run along the posterior surface of the heart, drain into the right atrium [10]. Approximately 96% of blood perfusing the left ventricular myocardium flows into the CS; therefore, measuring blood flow in the CS is considered a practical indicator reflecting the overall cardiac blood flow status [11]. Furthermore, patients with severe coronary artery disease often exhibit significant calcification at the coronary artery ostia, frequently making blood flow measurement using TEE difficult. In contrast, the CS is easily visualized in many cases, exhibits minimal turbulent flow components, and allows for accurate measurement at an angle nearly parallel to the echo beam [10]. The flow velocity pattern was similar to central vein, with systolic and diastolic antegrade waves, and a retrograde end diastolic wave [12].

In CPB-assisted CABG, TEE-measured CS blood flow reported significant increases in both peak velocity and VTI post-CPB compared to pre-CPB [1].

In this case, assuming no change in CS cross-sectional area (CSA) before and after CABG, the rate of increase in coronary perfusion flow before chest closure, compared to the flow after induction of anesthesia, was calculated to be 108% based on a comparison of the mean VTI × HR of CS blood flow. Furthermore, to account for the effect of increased CO due to higher catecholamine doses, we divided VTI × heart rate by CO and compared the resulting values at each time point. The calculated values were 120.28 after induction of anesthesia, 57.05 during heart displacement, 209.21 immediately after weaning from CPB, and 374.4 before chest closure. These results confirmed that, regardless of the effects on CO at each examination time point, similar to the absolute VTI values, coronary perfusion increased following coronary artery reconstruction and that coronary perfusion increased over time after weaning from CPB.

The VTI of the CS is included in the formula for calculating coronary blood flow: “Coronary flow ≈ CSA × VTI × HR.” Assuming that the CSA of the CS remains constant, an increase in this value of VTI indicates an increase in coronary blood flow. However, caution is required because variations in CSA size and shape may lead to inaccurate measurement of CS blood flow [13]. Additionally, appropriate placement of the sample volume may be difficult in cases with high heart rates [1]. On the other hand, the formula for calculating PI is “PI = (Vmax − Vmin) / Vmean,” and a decrease in this value indicates a decrease in peripheral vascular resistance in the coronary arteries. Since VTI and PI in CS reflect different aspects of blood flow, caution is required when interpreting these measurements. Furthermore, since preoperative PI values were not measured in this case, it cannot be definitively concluded that coronary vascular resistance improved as a result of CABG based solely on the postoperative values.

In addition, following a successful CABG procedure, maintaining high aortic pressure and low right atrial pressure is crucial for increasing coronary perfusion during anesthesia management [14].

Along with the PI, the VTI measurement using TEE to assess CS during CABG aids in evaluating the therapeutic efficacy of the procedure.

## Conclusions

When coronary blood flow was measured and compared as VTI on the CS before and after on-pump CABG, the values were highest in the following order: before sternal closure, immediately after weaning from the CPB, and after induction of anesthesia. Based on the above, we were able to evaluate the increase in coronary perfusion associated with coronary reconstruction and the gradual improvement in coronary perfusion following weaning from CPB.

In other words, evaluating blood flow not only in the primary artery but also in the collateral artery made it possible to easily assess perfusion in severely calcified coronary arteries, which could be utilized to help evaluate the effectiveness of treatment.
